# Sufficient Condition on the Fractional Integral for the Convergence of a Function

**DOI:** 10.1155/2013/428428

**Published:** 2013-12-15

**Authors:** Manuel A. Duarte-Mermoud, Norelys Aguila-Camacho, Javier A. Gallegos

**Affiliations:** ^1^Department of Electrical Engineering, University of Chile, Avenue Tupper 2007, Santiago de Chile 8370451, Chile; ^2^Advanced Mining Technology Center, Avenue Tupper 2007, Santiago de Chile 8370451, Chile

## Abstract

A sufficient condition on the fractional integral of the absolute value of a function is given in this paper, which allows to assure the convergence of the function to zero. This result can be useful to assure the convergence of a function when it is hard to know its exact evolution, but conditions on its fractional integral can be stated.

## 1. Introduction

Fractional calculus relates to the calculus of integrals and derivatives of orders that may be real or complex and has become very popular in recent years due to its demonstrated applications in many fields of science and engineering [1].

The nature of many systems makes them more precisely modeled using fractional differential equations. For instance, it can be mentioned the diffusion process founded in batteries [2], some heat transfer process [3], the effect of the frequency in induction machines [4], and the prediction of the groundwater flow [5], amongst others. In that sense, the stability of these systems have to be proved using techniques developed for fractional order systems.

The stability of fractional order nonlinear and time varying systems can be proved using the fractional order extension of Lyapunov's direct method [6]. Using this technique, however, is often a really hard task, since finding a Lyapunov candidate function is more complex in the fractional order case. Moreover, when a Lyapunov candidate function is found, most of the times its fractional derivative is only negative semidefinite, which assures the stability of the system but not the convergence of the states of the system.

For the integer order systems, Barbalat's Lemma [7] and some of its corollaries [8, 9] are used to prove the convergence of a function to zero based on some conditions on the integer integral of the function. However, in fractional order systems, it is usually more difficult to establish conditions on the integer integral of a function and consequently it can be tough to use the tools already mentioned.

Conditions on the fractional integral of a function can be more easily established in fractional order systems, and that is why it could be useful having a method to prove the convergence of a function to zero, based on some conditions on its fractional integral. This paper presents a lemma, which states a sufficient condition on a function and its fractional integral under which it can be assured that the function converges to zero.

The paper is organized as follows.  Section 2 presents some basic concepts about fractional calculus, facilitating the understanding of the ideas presented in this work.  Section 3 introduces Lemma 3, for assuring the convergence of a function to zero, some examples to support the result, and a potential application for Lemma 3. Finally, Section 4 presents the conclusions of the work.

## 2. Preliminaries

In what follows, some basic definitions related to fractional calculus are presented.

**Definition 1 (Riemann-Liouville fractional integral [1])**

The Riemann-Liouville fractional integral defined on a finite interval of the half-axis ℝ^+^ has the following form:

(1) $$\begin{array}{c} I_{a+}^{\alpha }x\left(t\right)=\frac{1}{\Gamma \left(\alpha \right)}\overset{t}{\underset{a}{\int }}\frac{x\left(\tau \right)}{{\left(t-\tau \right)}^{1-\alpha }}d\tau ,\;t>a,\;\;ℜ\left(\alpha \right)>0. \end{array}$$

There exist some different definitions for fractional derivatives. Equation (2) corresponds to the Caputo fractional derivative. To see other definitions and tables of fractional derivatives, see [1, 10].

**Definition 2 (Caputo fractional derivative [1])**

The Caputo fractional derivative of order *α* ∈ ℝ^+^ on the half-axis ℝ^+^ is defined as follows:

(2)   aCDtαx(t)=Iatn−α[Dnx(t)]=1Γ(n−α)∫atx(n)(τ)(t−τ)α−n+1dτ, t>a

with *n* = min⁡{*k* ∈ *ℕ*/*k* > *α*}, *α* > 0.

## 3. Sufficient Condition on the Fractional Integral for the Convergence of a Function to Zero

This section presents a new lemma, which allows to assure that a function converges to zero under some conditions on the function and its fractional integral.

### 3.1. The Main Lemma

**Lemma 3**

Let *x*(*t*) be a uniformly continuous function, *x*(·) : ℝ^+^ → ℝ. If there exists some 0 < *α* ≤ 1 such that

(3) $$\begin{array}{c} \underset{t\to \infty }{\lim}I_{t_{0}}^{\alpha }\left|x\left(t\right)\right|=0, \end{array}$$

then

(4) $$\begin{array}{c} \underset{t\to \infty }{\lim}x\left(t\right)=0. \end{array}$$

**Proof**

Let lim⁡_*t*→*∞*_ *x*(*t*) ≠ 0. Then there exists an infinite unbounded sequence {*t*_*i*_ : *i* ∈ *ℕ*} and *ε* > 0 such that

(5) $$\begin{array}{c} \left|x\left(t_{i}\right)\right|\geq \varepsilon >0,\;\forall t_{i}\in {\mathbb{R}}^{+}. \end{array}$$

Since *x*(*t*) is a uniformly continuous function, then for all *t*_*i*_ an associated interval [*t*_*i*_ − *δ*, *t*_*i*_ + *δ*], *δ* > 0, exists such that

(6) $$\begin{array}{c} \left|x\left(t_{i}\right)-x\left(t\right)\right|<\frac{\varepsilon }{2},\;\forall t\in \left[t_{i}-\delta ,t_{i}+\delta \right]. \end{array}$$

And using properties of the absolute value results, one has

(7) |x(t)|=|x(ti)−(x(ti)−x(t))|≥||x(ti)|−|(x(ti)−x(t))||,∀t∈[ti−δ,ti+δ].

Using (5) and (6) in (7) yields

(8) $$\begin{array}{c} \left|x\left(t\right)\right|\geq \frac{\varepsilon }{2},\;\forall t\in \left[t_{i}-\delta ,t_{i}+\delta \right]. \end{array}$$

The fractional integral of the absolute value of *x*(*t*) (Definition 1) over the interval [*t*_0_, *t*_*i*_] can be decomposed as

(9) It0α|x|(ti)=1Γ(α)∫t0ti−1|x(τ)|(ti−τ)1−αdτ+1Γ(α)∫ti−1ti−δ|x(τ)|(ti−τ)1−αdτ+1Γ(α)∫ti−δti|x(τ)|(ti−τ)1−αdτ.

Given that (|*x*(*τ*)|/(*t*_*i*_−*τ*)^1−*α*^) ≥ |*x*(*τ*)| for all *τ* ∈ [*t*_*i*_ − 1, *t*_*i*_] results in

(10) It0α|x|(ti)≥1Γ(α)∫t0ti−1|x(τ)|(ti−τ)1−αdτ+1Γ(α)∫ti−1ti−δ|x(τ)|dτ+1Γ(α)∫ti−δti|x(τ)|dτ≥1Γ(α)∫ti−δti|x(τ)|dτ.

Then using (8) in (10) yields

(11) It0α|x|(ti)≥1Γ(α)∫ti−δtiε2dτ=ε2Γ(α)(ti−(ti−δ))=εδ2Γ(α).

Given that the sequence {*t*_*i*_} is infinite, expression (11) contradicts the assumption that lim⁡_*t*→*∞*_ *I*_*t*_0__^*α*^|*x*(*t*)| = 0.

The following corollary can be useful when the existence of the limit of the fractional integral cannot be directly proved.

**Corollary 4**

Let *x*(*t*) be a uniformly continuous function, *x*(·) : ℝ^+^ → ℝ. If there exists some 0 < *α* ≤ 1 such that

(12) $$\begin{array}{c} I_{t_{0}}^{\alpha }\left|x\left(t\right)\right|=o\left(g\left(t\right)\right), \end{array}$$

where lim⁡_*t*→*∞*_ *g*(*t*) = 0 and *o*(·) according to [11], then

(13) $$\begin{array}{c} \underset{t\to \infty }{\lim}x\left(t\right)=0. \end{array}$$

**Proof**

Given that *I*_*t*_0__^*α*^|*x*(*t*)| = *o*(*g*(*t*)) and the fact that lim⁡_*t*→*∞*_ *g*(*t*) = 0 imply that lim⁡_*t*→*∞*_ *I*_*t*_0__^*α*^|*x*(*t*)| = 0. Then using Lemma 3 the conclusion is straightforward.

### 3.2. Some Examples

There are many functions which converge to zero and their fractional integral for some 0 < *α* ≤ 1 converges to zero. Some examples are presented below.

**Example 1**

Let *x*(*t*) = *t* ^−*γ*^, 0 < *γ* < 1. In this case, |*x*(*t*) | = *x*(*t*), and according to Kilbas et al. [1], the fractional integral *I*_*t*_0__^*α*^ *t* ^−*γ*^ results in

(14) $$\begin{array}{c} I_{t_{0}}^{\alpha }t^{-\gamma }=\frac{\Gamma \left(1-\gamma \right)}{\Gamma \left(1-\gamma +\alpha \right)}{\left(t-t_{0}\right)}^{\alpha -\gamma }. \end{array}$$

It can be seen from (14) that using some *α* < *γ* results in lim⁡_*t*→*∞*_ *I*_*t*_0__^*α*^ *t* ^−*γ*^ = 0, which is consistent with the result in Lemma 3.

**Example 2**

Let *x*(*t*) = *e* ^−*t*^. In this case, |*x*(*t*) | = *x*(*t*). There is no property which directly states the fractional integral of the function *x*(*t*) = *e* ^−*t*^, but it can be stated that

(15) $$\begin{array}{c} e^{-t}<t^{-\gamma },\;\forall \gamma \in \left(0,1\right). \end{array}$$

Based on (15) and according to [12] it can be concluded that

(16) $$\begin{array}{c} I_{t_{0}}^{\alpha }e^{-t}<I_{t_{0}}^{\alpha }t^{-\gamma },\;\forall \gamma \in \left(0,1\right),\;\;\forall t\geq t_{0},\;\;0<\alpha \leq 1. \end{array}$$

From Example 1 it is known that using some *α* < *γ* results in lim⁡_*t*→*∞*_ *I*_*t*_0__^*α*^ *t* ^−*γ*^ = 0. Consequently, from (16) it can be concluded that lim⁡_*t*→*∞*_ *I*_*t*_0__^*α*^ *e* ^−*t*^ = 0, which is consistent with the result in Lemma 3.

### 3.3. Potential Application

In what follows, a framework for a possible application of Lemma 3 to the convergence analysis of fractional adaptive systems is presented.

One of the parameterizations used for the representation of a plant for identification or adaptive control purposes is the one which has the form

(17) $$\begin{array}{c} y\left(t\right)=\theta^{T}\omega \left(t\right), \end{array}$$

where *ω*(*t*) ∈ ℝ^*n*^, *y*(*t*) ∈ ℝ and can be measured for all *t* ≥ *t*_0_, and *θ* is a constant vector in ℝ^*n*^ [8]. In identification problems, *θ* corresponds to the unknown parameters of the plant, while in control problems it represents the desired control parameter vector.

In both cases, an estimate $\hat{\theta }\left(t\right)\in {\mathbb{R}}^{n}$ of *θ* is used to generate an estimated output signal $\hat{y}\left(t\right)\in \mathbb{R}$. Defining $\phi \left(t\right)=\hat{\theta }\left(t\right)-\theta \left(t\right)$ and $e\left(t\right)=\hat{y}\left(t\right)-y\left(t\right)$, the error equation obtained is

(18) $$\begin{array}{c} e\left(t\right)=\phi^{T}\left(t\right)\omega \left(t\right). \end{array}$$

The purpose here is determining a rule for adjusting *ϕ*(*t*) (or equivalently $\hat{\theta }\left(t\right)$) from the knowledge of *ω*(*t*) and the error *e*(*t*), so that *e*(*t*) and in some cases *ϕ*(*t*) tend to zero asymptotically. It is well known [8] that the adaptive law for *ϕ* is given by

(19) $$\begin{array}{c} \dot{\phi }\left(t\right)=-e\left(t\right)\omega \left(t\right). \end{array}$$

Equation (19) together with (18) constitutes Error Model 1 [8].

However, the use of a fractional adaptive law instead of (19) has proved to be useful for reducing the effect of noise in the adaptive systems [13], and it can improve the convergence of the error to zero [14], so Fractional Error Model 1 has arisen, which is given by the following equations:

(20) e(t)=ϕT(t)ω(t)Dtα0Cϕ(t)=−e(t)ω(t),

where 0 < *α* < 1.

Many efforts are being made to demonstrate analytically the stability and convergence of the error *e*(*t*) in Fractional Error Model 1.

The Barbalat Lemma is used in the classic Error Model 1 to prove the convergence of the error *e*(*t*) to zero. However, the application of the Barbalat Lemma to the case of Fractional Error Model 1 (20) has been attempted without too much success, due to the difficulty in establishing conditions on the integer integral of the squared error ∫_*t*_0__^*t*^ *e* ^2^(*τ*)*dτ*.

Simulation studies suggest that, for the Fractional Error Model 1 (20), it holds that lim⁡_*t*→*∞*_ *I*_*t*_0__^*α*^ *e* ^2^(*t*) = 0. In that sense, Lemma 3 can be considered as a potential tool for proving the convergence of the error *e*(*t*) to zero in Fractional Error Model 1 (20), if it could be analytically proved that lim⁡_*t*→*∞*_ *I*_*t*_0__^*α*^ *e* ^2^(*t*) = 0.

## 4. Conclusions

A sufficient condition on the fractional integral of a uniformly continuous function *I*_*t*_0__^*α*^|*f*(*t*)| so that *f*(*t*) converges to zero has been presented in this paper. The result presented is valid for *α* ∈ (0,1]. It is expected that this result could be particularly useful in the analysis of adaptive systems with fractional order adaptive laws in order to prove the convergence of the control and/or identification errors to zero.
